# Outcome of patients with stage I immature teratoma after surveillance or adjuvant chemotherapy

**DOI:** 10.3389/fonc.2024.1330481

**Published:** 2024-02-02

**Authors:** Giuseppe Marino, Tommaso Grassi, Elena De Ponti, Serena Negri, Filippo Testa, Daniela Giuliani, Martina Delle Marchette, Cristina Dell’Oro, Diletta Fumagalli, Gianluca Donatiello, Giulia Besana, Liliana Marchetta, Cristina Maria Bonazzi, Andrea Alberto Lissoni, Fabio Landoni, Robert Fruscio

**Affiliations:** ^1^ Department of Medicine and Surgery, University of Milan-Bicocca, Milan, Italy; ^2^ Unit of Gynecology, Woman and Child Department, Istituto di Ricerca e Cura a Carattere Scientifico (IRCCS) San Gerardo, Monza, Italy

**Keywords:** immature teratoma of the ovary, germ cell tumor, chemotherapy, oncologic outcome, ovarian cancer

## Abstract

**Objective:**

Immature teratomas are rare malignant ovarian germ cell tumours, typically diagnosed in young women, where fertility-sparing surgery is the treatment of choice. The role of adjuvant chemotherapy in stage I disease remains controversial. We evaluated the impact of surveillance versus chemotherapy on the recurrence rate in stage I immature teratomas.

**Methods:**

We collected a single centre retrospective series of patients with stage I immature teratomas treated with fertility-sparing surgery at San Gerardo Hospital, Monza, Italy, between 1980 and 2019. Potential risk factors for recurrence were investigated by multivariate logistic regression.

**Results:**

Of the 74 patients included, 12% (9/74) received chemotherapy, while 88% (65/74) underwent surveillance. Median follow-up was 188 months. No difference in recurrence was found in stage IA/IB and IC immature teratomas [10% (6/60) vs. 28.6% (4/14) (P=0.087)], grade 1, grade 2, and grade 3 [7.1% (2/28) vs. 14.3% (4/28) vs. 22.2% (4/18) (p=0.39)], and surveillance versus chemotherapy groups [13.9% (9/65) vs. 11.1% (1/9)) (p = 1.00)]. In univariate analysis, the postoperative approach had no impact on recurrence. The 5-year disease-free survival was 87% and 90% in the surveillance and chemotherapy groups, respectively; the overall survival was 100% in both cohorts.

**Conclusions:**

Our results support the feasibility of surveillance in stage I immature teratomas. Adjuvant chemotherapy may be reserved for relapses. However, the potential benefit of chemotherapy should be discussed, especially for high-risk tumours. Prospective series are warranted to confirm our findings.

**What is already known on this topic:**

To date, no consensus has been reached regarding the role of adjuvant chemotherapy in stage I immature teratomas of the ovary. Some studies suggest that only surveillance is an acceptable choice. However, guidelines are not conclusive on this topic.

**What this study adds:**

No difference in terms of recurrence was observed between the surveillance and the adjuvant chemotherapy group. All patients who relapsed were successfully cured with no disease-related deaths.

**How this study might affect research, practice or policy:**

Adjuvant chemotherapy should be appropriately discussed with patients. However, it may be reserved for relapse according to our data.

## Introduction

Malignant ovarian germ cell tumours are rare malignancies accounting for approximately 5% of all ovarian cancers, with an estimated incidence of 3-4 cases/1,000,000 women in Europe (1). Immature teratomas represent approximately one-third of them and typically occur in young women, with a peak incidence between 15 and 30 years of age (1). Most patients are diagnosed with stage I disease and have an excellent prognosis (2). Disease grade and stage are two main prognostic factors (3). Given the young age at the diagnosis, the standard treatment is represented by fertility-sparing surgery with complete staging. In contrast, the need for adjuvant treatment is still controversial (4, 5). According to the National Comprehensive Cancer Network (NCCN) guidelines (4), patients diagnosed with stage IA grade 1 disease can avoid further treatments and undergo surveillance, while patients with stage I, grade 2 or 3 should receive adjuvant chemotherapy. However, due to the optimal prognosis with low rate of recurrence and the potential side effects of the therapy (6–11), the European Society for Medical Oncology (ESMO) guidelines suggest that close surveillance may also be considered in stage IA grade 2 or 3 and stage IB–IC, and chemotherapy reserved as salvage therapy for recurrence (5).

We report a large retrospective case series of post-pubertal patients with stage I, any grade, immature teratomas treated at our Institution. The primary aim of this study was to evaluate the impact of adjuvant chemotherapy or surveillance on the recurrence rate. Disease-free and overall survival were also assessed.

## Methods

### Patients characteristics

Patients with pathologically confirmed stage I pure immature teratoma treated at San Gerardo Hospital, Monza, between 1980 and 2019 were screened for inclusion. All cases were reviewed by a dedicated pathologist who categorised the tumours into three grades (3). The tumour stage was defined according to the 2014 Federal International Federation of Gynecology Oncology (FIGO) classification for ovarian cancer (12), adapting our cases previously diagnosed to this updated version.

Inclusion criteria were post-pubertal age (intended as post-menarche period) and treatment with primary fertility-sparing surgery, defined as preservation of the uterus and at least one adnexa. The type of ovarian surgery was defined as unilateral salpingo-oophorectomy (removal of the affected ovary and the ipsilateral fallopian tube with the preservation of the contralateral adnexa) or cystectomy (enucleation of the cystic lesion with preservation of both the adnexa). In bilateral cysts, fertility was preserved by performing a unilateral salpingo-oophorectomy + cystectomy. Complete surgical staging procedures, defined as omentectomy, peritoneal washing, and peritoneal biopsies, were also performed at the time of diagnosis or during surgical restaging. If primary surgery was not performed at our Institution, surgical restaging was performed within 90 days from the diagnosis and considered a complete staging. Despite these attempts, incomplete surgical staging was observed in most patients. All patients who did not undergo surgical staging were staged with imaging techniques, such as computed tomography (CT). Patient follow-up has changed over decades. From 1980 to 2000, the follow-up visit included a gynecologic examination with transvaginal ultrasound and alpha-fetoprotein measurement, CT, and laparoscopy ± biopsies. In the last two decades, with the improvement of imaging techniques, routine second-look laparoscopy was almost abandoned in the absence of suspected recurrence. Relapse was confirmed after a histological sampling obtained by biopsy or surgery. Follow-up was performed every 3 months for the first 2 years, then every 6 months until the fifth year, then yearly (5). Patients with less than 24 months of follow-up were excluded. Ethical approval from Comitato Etico Brianza was obtained (3930).

### Statistical analysis

For descriptive statistics, frequencies and proportions were used for categorical variables, while for continuous variables, means or medians were used with standard deviation or minimum-maximum range, respectively. Continuous variables were compared using the Wilcoxon rank sum test, while proportions were compared using the Chi-square test or Fisher’s exact test. All p values are two-sided and were considered statistically significant if p < 0.05.

A multivariate logistic regression was performed to assess the event of disease recurrence and possible independent associations between patient, disease and treatment variables. Logistic regression was used for the analysis since the endpoint was binary. Disease-free survival curves were estimated with the Kaplan-Meier method. Stata Software 9.0 (Stata Corporation, College Station, TX, USA) was used for the analysis.

## Results

**Figure 1:**
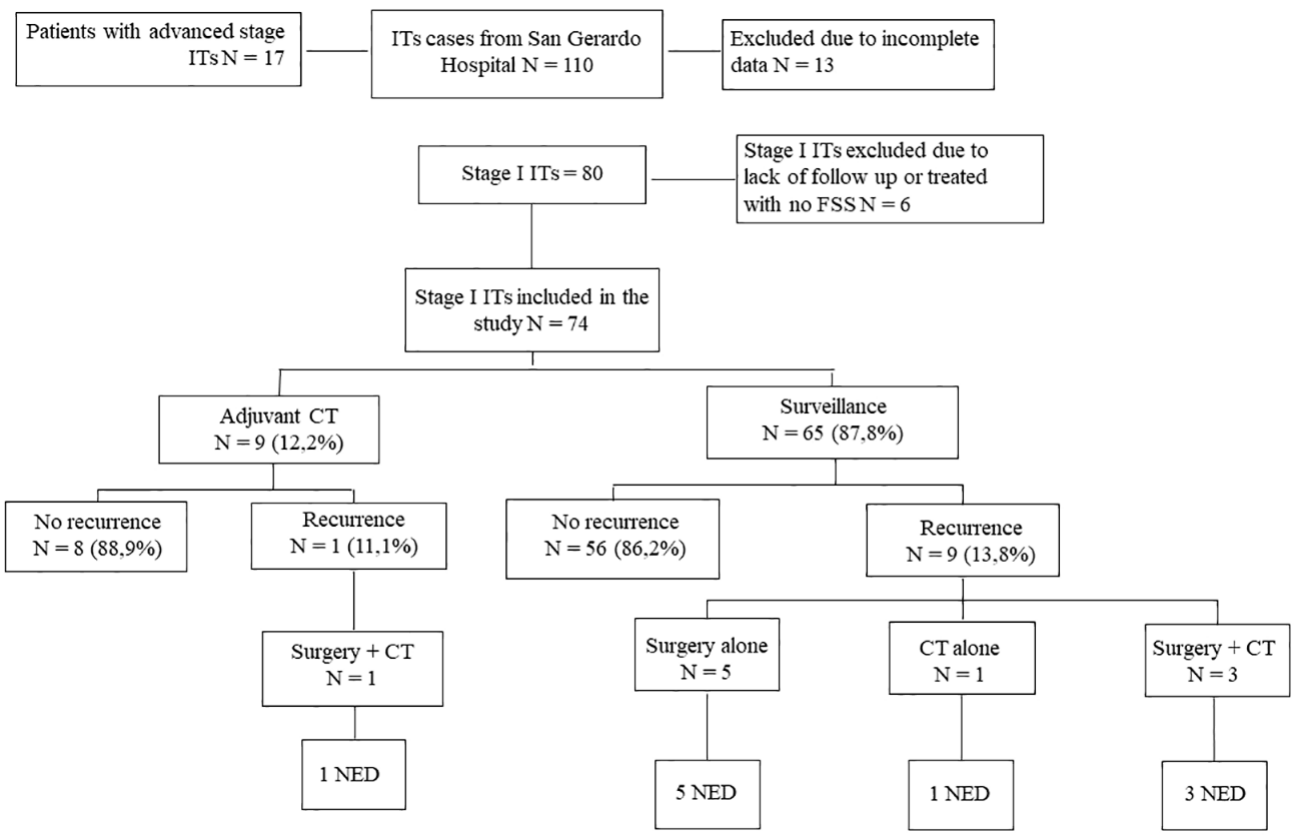
Study Flowchart. Flow chart relative to management and outcome of stage I ITs patients (from San Gerardo Hospital, Monza, between 1980 and 2019). ITs, Immature Teratomas; CT, Chemotherapy; NED, No Evidence of Disease.

Between 1980 and 2019, 110 post-pubertal patients with pure immature teratomas were referred to our Institution. Eighty of them had a stage I disease, as reported in **Figure 1**. Six patients were lost at follow-up, and 74 were included in the analysis. Patients’ characteristics are shown in **Supplementary Tables 1**, **2A**.

The median age at diagnosis was 27 years. Eighty percent of patients were stage IA (59/74), 1% were stage IB (1/74), and 19% stage IC (14/74). The rate of patients with grade 1, 2, and 3 was 38% (28/74), 38% (28/74), and 24% (18/74), respectively. Seventy-two percent of patients underwent unilateral salpingo-oophorectomy (53/74), whereas a cystectomy was performed in 28% (21/74). Seventy-four percent of patients (55/74) underwent a laparotomy, while 25% (19/74) underwent a laparoscopic procedure. Laparotomy was the preferred approach up to 2000, while 40% (10/25) of the procedures performed after 2000 were laparoscopic. Only 23% of patients (17/74) underwent complete surgical staging, while 77% (57/74) of cases did not, and further surgical staging was waived.

As shown in **Table 1**, 12% of patients (9/74) underwent adjuvant chemotherapy. Eight received 3 cycles of bleomycin/etoposide/cisplatin regimen, and only one received 3 cycles of bleomycin/vincristine/cisplatin schedule. Surveillance alone was recommended in 88% of patients (65/74). Among the 9 patients who received adjuvant chemotherapy 55.6% had stage IC disease while 44.4% had stage IA/B (p 0.010); also, 55.6% had grade 3while 33.3% had grade 2 and 11.1% grade 1 (p 0.058) (**Table 1**; **Supplementary Table 5**). A lower median age was observed in patients treated with chemotherapy [p value = 0.052]. Among patients who underwent adjuvant chemotherapy, 7 were treated before 2000, while 2 in the last two decades, favouring a “wait and see behaviour” (13). The type of ovarian surgery and the complete surgical staging did not influence the postoperative treatment (**Table 1**; **Supplementary Tables 3, 4**).

**Table 1:** Patients’ characteristics according to post-operative treatment.

Post-operative treatment	Surveillance (n=65)	Chemotherapy (n=9)	p value
**Median age** (min-max)	28	18	0.052
**Decades of treatment**			0.386
1980-1989 1990-1999 2000-2009 2010-2019	11 (16.9%) 30 (46.2%) 20 (30.8%) 4 (6.2%)	3 (33.3%) 4 (44.4%) 1 (11.1%) 1 (11.1%)	
**Stage**			0.010
IA + IB IC	56 (86.2%) 9 (13.8%)	4 (44.4%) 5 (55.6%)	
**Grade**			0.058
Grade 1 Grade 2 Grade 3	27 (41.5%) 25 (38.5%) 13 (20.0%)	1 (11.1%) 3 (33.3%) 5 (55.6%)	
**Type of surgery**			0.431
Cystectomy Unilateral Salpingo-Oophorectomy	20 (30.8%) 45 (69.2%)	1 (11.1%) 8 (88.9%)	
**Complete staging**			0.675
Yes No	16 (24.6%) 49 (75.4%)	1 (11.1%) 8 (88.9%)	
**Relapse**			1.00
	9 (13.9%)*	1 (11.1%)*	

*column percentage.

**Figure 2:**
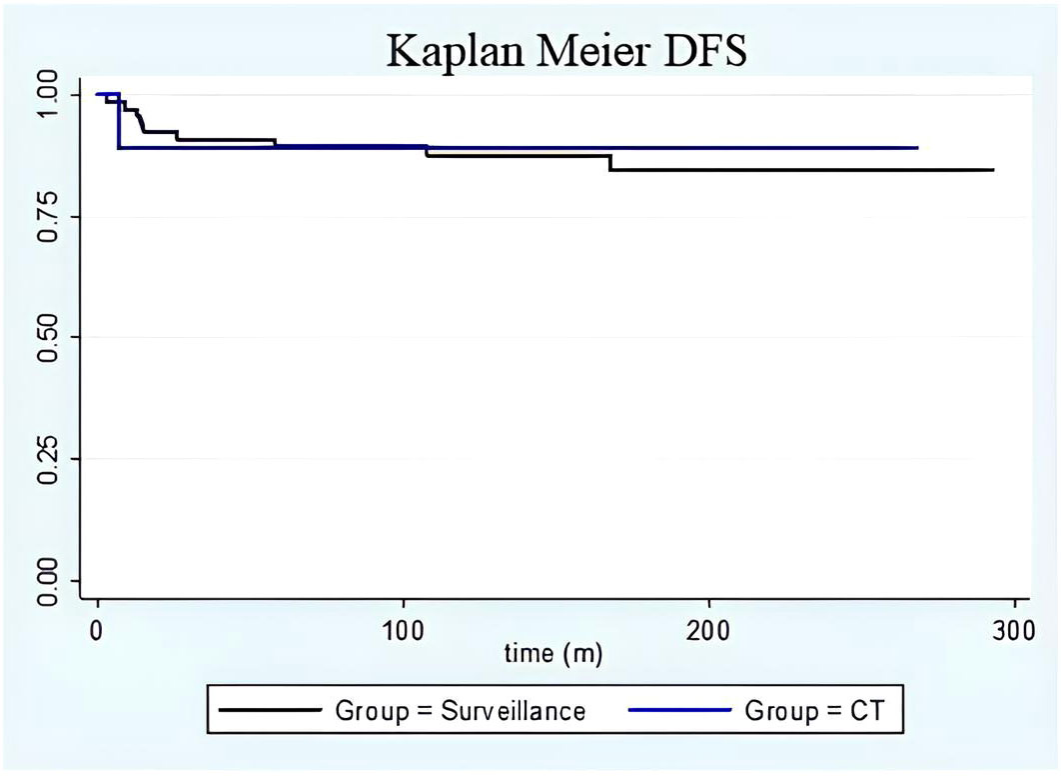
DFS Kaplan Meier. Kaplan-Meier curve on disease-free survival in the surveillance group (black line) versus adjuvant chemotherapy group (blue line). No differences were found between the groups in terms of disease-free survival. Moreover, time-to-relapse between the groups did not show any differences.

Oncologic outcomes are summarised in **Table 2**; **Supplementary Table 2B**. Among 10 relapsing patients, 1 (10%) received chemotherapy, while 9 (90%) underwent surveillance; the same percentages were observed among patient who did not have a relapse (12.5% and 87.5%, respectively, p: 1.00) (**Figure 2**). Six relapses were found in the IA+IB stage group (6/10 = 60%) and 4 in the IC stage group (4/10 = 40%) [p value = 0.087]. Among relapsed patients 20% had grade 1, 40% grade 2 and 40% grade 3 [p value = 0.390]. The recurrence rate was not different among patients who underwent a different surgical approach or type of ovarian surgery (**Table 2**; **Supplementary Table 3**). Moreover, no significant difference was observed in terms of recurrence rate among patients who underwent complete staging at the time of primary surgery (4/10 = 40.0%) and patients who did not (6/10 = 60.0%) [p value 0.224] (**Table 2**; **Supplementary Table 4**).

**Table 2:** Oncologic outcomes.

Relapse	Yes (N = 10)	No (N = 64)	p value
**Median age (min-max)**	22.5 (12-39)	27.5 (11-42)	0.304
**Stage**			0.087
IA+IB IC	6 (60.0%) 4 (40.0%)	54 (84.4%) 10 (15.6%)	
**Grade**			0.390
Grade 1 Grade 2 Grade 3	2 (20.0%) 4 (40.0%) 4 (40.0%)	26 (40.6%) 24 (37.5%) 14 (21.9%)	
**Surgical approach**			0.770
Laparotomy Laparoscopy Non available	7 (70.0%) 3 (30.0%) 0 (0%)	48 (75.0%) 14 (21.9%) 2 (3.1%)	
**Type of surgery**			0.715
Cystectomy Unilateral Salpingo-Oophorectomy	2 (20.0%) 8 (80.0%)	19 (29.7%) 45 (70.3%)	
**Post-surgical approach**			1.00
Surveillance Chemotherapy	9 (90.0%) 1 (10.0%)	56 (87.5%) 8 (12.5%)	
**Complete staging**			0.224
Yes No	4 (40.0%) 6 (60.0%)	13 (20.3%) 51 (79.7%)	

Among patients who received chemotherapy, the one who experienced a recurrence had stage IC grade 3 immature teratoma treated with a laparoscopic unilateral salpingo-oophorectomy and 3 subsequent cycles of bleomycin/etoposide/cisplatin. She developed an umbilical recurrence 7 months after the diagnosis, and she was successfully treated with surgery followed by two more cycles of bleomycin/etoposide/cisplatin. For the surveillance group, the time to relapse was between 3 and 168 months after surgery (median time 46 months; **Figure 2**). The characteristics of patients who experienced recurrence are summarised in **Supplementary Table 6**: 3 patients relapsed only in the contralateral ovary, 5 presented with peritoneal metastases (3 had only pelvic peritoneal involvement), and one patient experienced peritoneal and lymphatic recurrence. All patients were successfully treated with surgery ± chemotherapy. All patients were alive at the time of the last follow-up and with no evidence of disease. Only one patient was diagnosed with a second recurrence that was successfully treated. She had a stage IC2 (capsule ruptured before surgery) grade 3 disease at the time of the diagnosis; she underwent a complete surgical staging, and no adjuvant treatment was advised. Her first relapse was diagnosed nine months after surgery: she underwent 5 cycles of bleomycin/etoposide/cisplatin for diffuse intraperitoneal and visceral lesions, with complete remission. One year later, she developed a second localised relapse in the pouch of Douglas that was surgically removed, and a second-line adjuvant chemotherapy with Paclitaxel/Ifosfamide/Cisplatin was recommended (patient 9 in **Supplementary Table 6**).

### Univariate analysis

Univariate analyses was performed to evaluate the prognostic role of the different clinicopathological variables on the recurrence rate (**Table 3**).

**Table 3:** Association between clinical characteristics and relapse (univariate analysis).

	Univariate		
	OR	C.I. 95%	P value
**Age at diagnosis** (Years)	0.95	0.88 – 1.04	0.287
**Grade** G3 vs. G1-G2	2.38	0.59 – 9.63	0.224
**Stage** IC vs. IA-IB	3.60	0.86 – 15.1	0.080
**Adjuvant Chemotherapy** Yes vs. No	0.78	0.09 – 6.98	0.822

OR, Odds ratio; C.I., Confidence interval; G1, grade 1; G2, grade 2; G3, grade 3.

No factors showed a statistically significant impact on the relapse rate, and adjuvant chemotherapy did not show a protective effect. Stage of disease showed an Odd Ratio of 3.60 (CI: 95% 0.86 –15.1; stage IC vs. IA-IB), however, it did not reach statistical significance (p = 0.08).

The 5-year disease-free survival was 87.4% and 90.0% for patients who underwent surveillance and adjuvant chemotherapy, respectively (**Figure 2**). During follow-up, no patient died of the disease or by any means, with a disease-specific and overall survival of 100% for the whole cohort.

## Discussion

### Summary of main results

Patients in our study who underwent active surveillance did not show any worse oncological outcome when compared to those treated with chemotherapy. Disease-free survival was similar at five years between the two groups, suggesting that adjuvant chemotherapy neither improved oncologic outcome nor moved time-to-relapse further forward in the study population. Additionally, all patients who relapsed were successfully cured, with no disease-related deaths occurring during follow-up. There were some differences in the relapse rates between different grades (7.1% in grade 1 ITs, 14.3% in grade 2, and 22.2% in grade 3) and stages (28.6% for IC, while it was 10% in stage IA or IB). However, due to the small sample size, the present study was probably unable to reach statistical significance. Additionally, in our cohort, the absence of surgical staging was not a critical risk factor for a worse oncologic outcome, differently from other evidence (14).

Of note, these results corroborate the opinion of those who argue that chemotherapy can be omitted in the standard therapeutic approach for stage I disease.

### Results in the context of published literature

Studies conducted between the 1970s and the 1990s suggest that patients with early-stage grade 2-3 disease should receive adjuvant chemotherapy because of their high risk of recurrence and the survival benefit after chemotherapy (3, 15–18). However, chemotherapy may cause long-term toxicities, such as secondary malignancies after etoposide exposure, bleomycin’s pulmonary effects, and platinum neurotoxicity (6–11, 19). Additionally, the risk of chemotherapy-induced amenorrhea is higher with the increase in dosage and the number of therapy cycles (5), although the standard bleomycin/etoposide/cisplatin schedule seems not to impair the ovarian reserve (20).

Recently, echoing the positive experiences of surveillance in the paediatric population affected by immature teratomas (21, 22) and the established practice of avoiding adjuvant chemotherapy in some male germ cell tumours (23), some authors suggested active surveillance as an alternative to adjuvant chemotherapy in patients with post-pubertal stage I immature teratoma, reserving chemotherapy for patients with recurrent disease (5, 24–29).

An Italian multicentre study (26) found an optimal long-term prognosis in 28 patients with stage I pure immature teratomas with post-surgery surveillance and recommended chemotherapy in case of recurrence or in the presence of a yolk sac tumour component because it worsens the prognosis. Recently, Bergamini et al. (24) retrospectively analysed a large group of 108 patients with stage I pure immature teratomas who underwent surveillance or adjuvant chemotherapy after fertility-sparing surgery and were followed up at Charing Cross Hospital, London, United Kingdom, and in Italy. Stage IA, IB, and IC were respectively 66, 3, and 39 on a cohort of 108 patients. Twenty-five percent received adjuvant chemotherapy, while 75% underwent surveillance only. The recurrence rate was not different between the two groups [7.4% (2/25) vs. 11.1% (9/81), respectively (p 0.65)]. Moreover, all patients who relapsed were successfully cured at the time of recurrence, except for one who did not adhere to the recommended close follow-up procedures. Thus, they suggest surveillance as a replacement for adjuvant chemotherapy in stage I immature teratomas of any grade in the adult setting, reserving systemic treatment only for recurrent disease. Bergamini et al. (24) also found that tumour grade and complete surgical staging were the only independent prognostic factors for worse disease-free survival. In 1994, D. M. O’Connor and H. J. Norris identified the tumour grade as one of the most important risk factors for relapse in these patients (30), showing a recurrence rate of 70% in grade 3 disease and 18% in grade 2 disease. A significant association between grade and risk of recurrence is extensively reported in the literature (30–32), further confirmed by Pashankar et al. (27) and Zhao et al. (33).

Surgical staging is one of the cornerstones in the management of these patients, reported in guidelines as mandatory (4, 5, 14). Also, it’s common in clinical practice to do a second-step surgery in patients not properly staged and results from Bergamini et al. (24) confirmed this crucial aspect. Indeed, a selection bias may have occurred in our population, as we retrospectively analysed only early-stage disease, so further evidence from prospectively-collected data is warranted to clarify these findings.

Additionally, stage represents one of the most important and well-known prognostic factors for poor oncologic outcomes (34–36). However, Bergamini et al. (24) did not find a significant correlation between the substage of stage I disease and worsening outcomes. Despite our data showed that the prognostic factor most associated with relapse was the stage of disease [Odd Ratio = 3.60 (CI 95% 0.86 – 15.1)], no significance was reached. Due to the low rate of relapse in our population, a multivariate analysis appeared to be not feasible from a statistical point of view, limiting in part the statistical strength of our study, even if it would not have showed significant differences.

Finally, all patients who developed a diffuse relapse of disease, which required extensive surgery and subsequent chemotherapy, had a high-risk disease at the time of the diagnosis (grade 3 or stage IC or both - **Supplementary Table 6**). Therefore, we suggest carefully evaluating adjuvant treatment, discussing individual cases in multidisciplinary meetings, and adequate counselling with the patient, especially for high-risk tumours.

### Strengths and limitations

The main limitations of the present study are its retrospective design, the small sample size of the population analysed, and the low number of patients with high-risk disease. The small sample size limits the study’s power on reaching a real difference between the two cohorts, remarked also by the low rate of events that have limited the possibility of performing a multivariate analysis. Also, the low percentage of complete surgical staging represents a further limit. Nevertheless, this is the largest European single-centre case series reported in the literature based on a population of patients with pure ovarian immature teratoma. An advantage of being monocentric is the homogeneity of patient treatment. We found no significant differences between the postoperative treatment of patients, in terms of chemotherapy or surveillance, in the four decades considered in the study.

### Implications for practice and future data

Given the limited data available on this topic, our research highlights and agrees with other Authors on the central role of surveillance in stage I immature teratomas, suggesting that adjuvant chemotherapy may be reserved for relapses. Future studies, in particular prospective collections, are required to confirm the impact of surveillance on disease recurrence.

## Conclusions

Our data confirm that stage I immature teratomas are characterised by an excellent prognosis in terms of disease recurrence, as reported in the literature (2, 20, 26, 32).

As previously reported, we can conclude that adjuvant chemotherapy may be omitted in this selected population after extensive counselling, reserving it for disease relapse. However, especially for high-risk stage I tumours (stage IC and grade 3), adjuvant treatment should be discussed with the patient on an individual basis. No independent prognostic factors were found to be statistically significant in predicting relapse.

In our cohort, active surveillance resulted as a safe alternative to adjuvant chemotherapy for the postoperative management of stage I ovarian immature teratomas. Nevertheless, prospective series are needed to confirm our findings.

## Data availability statement

The raw data supporting the conclusions of this article will be made available by the authors, without undue reservation.

## Ethics statement

The studies involving humans were approved by IRB Comitato Etico Brianza, IRCCS San Gerardo dei Tintori, Monza, Italy. The studies were conducted in accordance with the local legislation and institutional requirements. The participants provided their written informed consent to participate in this study.

## Author contributions

GM: Investigation, Writing – original draft. TG: Investigation, Writing – original draft. EDP: Methodology, Writing – review & editing. SN: Investigation, Writing – review & editing. FT: Investigation, Writing – review & editing. DG: Conceptualization, Investigation, Writing – original draft. MDM: Investigation, Writing – review & editing. CD’O: Investigation, Validation, Writing – review & editing. DF: Investigation, Writing – review & editing. GD: Investigation, Writing – review & editing. GB: Investigation, Writing – review & editing. LM: Investigation, Writing – review & editing. CB: Conceptualization, Investigation, Methodology, Supervision, Writing – review & editing. AL: Conceptualization, Supervision, Writing – review & editing. FL: Supervision, Writing – review & editing. RF: Conceptualization, Investigation, Project administration, Supervision, Writing – original draft.
