# Supplementary material for: Outcome of patients with stage I immature teratoma after surveillance or adjuvant chemotherapy
Source: Front Oncol. 2024 Feb 2;14:1330481. doi: 10.3389/fonc.2024.1330481 (PMC10869612; doi:10.3389/fonc.2024.1330481)
Supplement: Supplementary file 1 [file Table_1.docx]

**Supplementary Tables**

| **Supplementary Table 1. Demographic and clinical characteristics of patients included in the analysis (N = 74)** | | |
| --- | --- | --- |
|  | **Average**  **(± Standard Deviation)** | **Median**  **(min-max)** |
| **Age at diagnosis** | 26.5 (± 8.0) | 27 (11-42) |
| **Grade**  Grade 1  Grade 2  Grade 3 | 28 (37.8%)  28 (37.8%)  18 (24.3%) | |
| **Stage**  IA  IB  IC | 59 (79.7%)  1 (1.4%)  14 (18.9%) | |
| **Decades of surgery**  1980-1989  1990-1999  2000-2009  2010-2019 | 14 (18.9%)  34 (45.9%)  21 (28.4%)  5 (6.8%) | |
| **Surgical approach**  Laparotomy  Laparoscopy  Non available | 55 (74.3%)  17 (23.0%)  2 (2.7%) | |
| **Type of surgery**  Cystectomy  Unilateral Salpingo-Oophorectomy | 21 (28.4%)  53 (71.6%) | |
| **Complete staging**  No  Yes | 57 (77.0%)  17 (23.0%) | |
| **Post-surgical approach**  Surveillance  Cchemotherapy | 65 (87.8%)  9 (12.2%) | |
| **Follow up (months)** | **Average (± DS)**  191.8 (± 78.6) | **Median**  **(min-max)**  188.5 (24.0-369.0) |

| **Supplementary Table 2A. Tumors’ stage (according to FIGO classification) and grade (according to WHO classification)** | | | |
| --- | --- | --- | --- |
| **Staging** | **Grading - Number (%) of patients** | | |
|  | Grade 1  (N = 28) | Grade 2  (N = 28) | Grade 3  (N = 18) |
| IA  (N = 59) | 24 (32.4%) | 22 (29.7%) | 13 (17.6%) |
| IB  (N = 1) | 1 (1.3%) | 0 | 0 |
| IC  (N = 14) | 3 (4.1%) | 6 (8.1%) | 5 (6.8%) |
| Abbreviations. FIGO = International Federation of Gynecology and Obstetrics; WHO = World Health Organization | | | |

| **Supplementary Table 2B. Recurrence rate based on tumors’ stage (according to FIGO classification) and grade (according to WHO classification)** | | | | |
| --- | --- | --- | --- | --- |
| **Staging** | **Grading - Recurrence rate** | | | |
|  | Grade 1  (N=28) | Grade 2  (N=28) | Grade 3  (N=18) | **p value** |
| IA  (N = 59) | 1/24 (4.2 %) | 3/22 (13.6%) | 2/13 (15.4%) | 0.418 |
| IB  (N = 1) | 0/1 (0%) | - | - | Non available |
| IC  (N = 14) | 1/3 (33.3%) | 1/6 (16.7%) | 2/5 (40%) | 0.775 |
| Abbreviations. FIGO = International Federation of Gynecology and Obstetrics; WHO = World Health Organization | | | | |

| **Supplementary Table 3. Patients’ characteristics and type of ovarian surgery** | | | |
| --- | --- | --- | --- |
| **Type of ovarian surgery** | **Cystectomy**  (n=21) | **Unilateral Salpingo-Oophorectomy**  (n=53) | **p value** |
| **Median age** (min-max) | 24.9 | 27.1 | 0.239 |
| **Decades of treatment**  1980-1989  1990-1999  2000-2009  2010-2019 |  | | 0.074 |
|  | 1 (4.8%)  9 (42.8%)  8 (38.1%)  3 (14.3%) | 13 (24.5%)  25 (47.2%)  13 (24.5%)  2 (3.8%) |  |
| **Stage**  IA + IB  IC |  | | 0.774 |
|  | 18 (30.0%)  3 (21.4%) | 42 (70.0%)  11 (78.6%) |  |
| **Grade**  Grade 1  Grade 2  Grade 3 |  | | 0.025 |
|  | 13 (46.4%)  6 (21.4%)  2 (11.1%) | 15 (53.6%)  22 (78.6%)  16 (88.9%) |  |
| **Post-operative treatment**  Surveillance  Chemotherapy |  | | 0.431 |
|  | 1 (11.1%)  20 (30.8%) | 8 (88.9%)  45 (69.2%) |  |
| **Relapse** | 2 (9.5%)* | 8 (15.1%)* | 0.209 |
| *column percentage | | | |

| **Supplementary Table 4. Surgical staging** | | | |
| --- | --- | --- | --- |
| **Complete staging** | **Yes**  (n=17) | **No**  (n=57) | **p value** |
| **Median age** (min-max) | 27 (11-39) | 27 (12-42) | 0.862 |
| **Decades of treatment**  1980-1989  1990-1999  2000-2009  2010-2019 | 3 (21.4%)  8 (23.5%)  4 (19.1%)  2 (40.0%) | 11 (78.6%)  26 (76.5%)  17 (80.9%)  3 (60.0%) | 0.817 |
| **Stage**  IA + IB  IC | 12 (20.3%)  5 (35.7%) | 48 (79.7%)  9 (64.3%) | 0.289 |
| **Grade**  Grade 1  Grade 2  Grade 3 | 6 (21.4%)  5 (17.9%)  6 (33.3%) | 22 (78.6%)  23 (82.1%)  12 (66.7%) | 0.527 |
| **Surgical Approach**  Laparotomy  Laparoscopy  Non available | 16 (29.1%)  1 (5.9%)  0 (0%) | 39 (70.9%)  16 (94.1%)  2 (100%) | 0.118 |
| **Type of ovarian surgery**  Unilateral Salpingo-Oophorectomy  Cystectomy | 13 (24.5%)  4 (19.1%) | 40 (75.5%)  17 (80.9%) | 0.763 |
| **Post-operative treatment**  Surveillance  Chemotherapy | 16 (24.6%)  1 (11.1%) | 49 (75.4%)  8 (88.9%) | 0.675 |
| **Relapse**  No  Yes | 13 (20.3%)  4 (40.0%) | 51 (79.7%)  6 (60.0%) | 0.224 |

| **Supplementary Table 5. Patients’ characteristics and post operative treatment** | | | | |
| --- | --- | --- | --- | --- |
| **Post operative treatment** | **Surveillance** (n=65) | **Chemotherapy**  (n=9) | **Relapses on Surveillance** | **Relapses on Chemotherapy** |
| **Decades of treatment**  1980-1989  1990-1999  2000-2009  2010-2019 |  | | | |
|  | 11 (78.6%)  30 (88.2%)  20 (95.2%)  4 (80.0%) | 3 (21.4%)  4 (11.8%)  1 (4.8%)  1 (20.0%) | 2 (14.3%)  3 (8.8%)  3 (14.3%)  1 (20%) | 0 (0%)  0 (0%)  1 (4.8%)  0 (0%) |
| **Stage and grades**  IA-Grade 1  IA-Grade 2  IA-Grade 3  IB-Grade 1  IC-Grade 1  IC-Grade 2  IC-Grade 3 | 24 (100%)  21 (95.5%)  10 (76.9%)  1 (100%)  2 (66.7%)  4 (66.7%)  3 (60.0%) | 0 (0%)  1 (4.5%)  3 (23.1%)  0 (0%)  1 (33.3%)  2 (33.3%)  2 (40.0%) | 1/24 (4.1%)  3/21 (14.3%)  2/10 (20%)  0 (0%)  1/2 (50%)  1/4 (25%)  1/3 (33.3%) | 0 (0%)  0/1 (0%)  0/3 (0%)  0 (0%)  0/1 (0%)  0/2 (0%)  1/2 (50%) |
|  |  |  |  |  |

| **Supplementary Table 6. Characteristics of patients with recurrence and relapse features** | | | | | | | | | | |
| --- | --- | --- | --- | --- | --- | --- | --- | --- | --- | --- |
|  | **Age at diagnosis** | **Stage**  **and**  **Grade** | **Type of surgery**  **and**  **Complete staging** | **LPT/LPS** | **CT** | **Site of relapse** | **Histotype of relapse** | **Time to relapse** | **Treatment of relapse** | **Vital status** |
| **1** | 16 | IA  G1 | Cy  Yes | LPT | No | Right ovary, right broad ligament | IT G3 | 108 | Surgery + CT | NED |
| **2** | 12 | IA  G2 | USO  No | LPT | No | Hepato-diafragmatic peritoneum. Peritectal tissue, lymphnodes, omentum | IT G1 | 13 | Surgery alone | NED |
| **3** | 21 | IA  G2 | Cy  Yes | LPS | No | Right ovary | IT G1 | 14 | Surgery alone | NED |
| **4** | 15 | IA  G2 | USO  No | LPT | No | Pelvic peritoneum | Immature glia implants | 15 | Surgery + CT | NED |
| **5** | 24 | IA  G3 | USO  No | LPT | No | Left ovary | IT G1 islet in the context of mature teratoma | 26 | Surgery alone | NED |
| **6** | 31 | IA  G3 | USO  No | LPT | No | Douglas pouch, vescico-uterine peritoneum, right parieto-colic gutter, mesenter, small and large bowels | IT G3 | 3 | Surgery + CT | NED |
| **7** | 19 | IC  G1 | USO  Yes | LPT | No | Left uterosacral ligament | IT G1 | 168 | Surgery alone | NED |
| **8** | 31 | IC  G2 | USO  No | LPS | No | Left ovary | IT G1 | 58 | Surgery alone | NED |
| **9** | 39 | IC  G3 | USO  Yes | LPT | No | Wide peritoneal thickening, Douglas pouch, Glisson capsule | Glia immatura diffusamente infiltrante | 9 | CT + surgery + CT | NED |
| **10** | 32 | IC  G3 | USO  No | LPS | Yes | Umbelical nodule | Immature glia in the context of mature glia | 7 | Surgery + CT | NED |
| Abbreviations. LPT = Laparotomy; LPS = Laparoscopy; CT = Chemotherapy; Cy = Cystectomy; USO = Unilateral Salpingo-Oophorectomy; IT = Immature teratoma; NED = No evidence of disease; AWD = Alive with disease | | | | | | | | | | |
